# Precise planning based on 3D-printed dry-laboratory models can reduce perioperative complications of laparoscopic surgery for complex hepatobiliary diseases: a preoperative cohort study

**DOI:** 10.1186/s12893-024-02441-z

**Published:** 2024-05-11

**Authors:** Wei-Feng Yao, Xiao-Kun Huang, Tian-Wei Fu, Lei Jin, Cheng-Fei Du, Zhen-Yu Gao, Kai-Di Wang, Mu-Gen Dai, Si-Yu Liu, Jun-Wei Liu, Cheng-Wu Zhang, Lei Liang, Dong-Sheng Huang

**Affiliations:** 1 Department of Clinical Medicine, Medical College of Soochow University, Suzhou, China; 2 General Surgery, Cancer Center, Department of Hepatobiliary & Pancreatic Surgery and Minimally Invasive Surgery, Affiliated People’s Hospital, Zhejiang Provincial People’s Hospital, Hangzhou Medical College, Hangzhou, Zhejiang China; 3 Department of Postgraduate Training, Base Alliance of Wenzhou Medical University, Wenzhou, Zhejiang China; 4 Department of the Second School of Clinical Medicine, Zhejiang Chinese Medical University, Hangzhou, Zhejiang China; 5 Department of Gastroenterology, The Fifth Affiliated Hospital of Wenzhou Medical University, Lishui, Zhejiang China; 6 Department of Laboratory Medicine, The Key Laboratory of Imaging Diagnosis and Minimally Invasive Interventional Research of Zhejiang Province, Zhejiang University Lishui Hospital, Lishui, Zhejiang China

**Keywords:** 3D-printed model, Hepatobiliary disease, Laparoscopic surgery, Postoperative complications, Intraoperative navigation

## Abstract

**Background & aims:**

Complications after laparoscopic liver resection (LLR) are important factors affecting the prognosis of patients, especially for complex hepatobiliary diseases. The present study aimed to evaluate the value of a three-dimensional (3D) printed dry-laboratory model in the precise planning of LLR for complex hepatobiliary diseases.

**Methods:**

Patients with complex hepatobiliary diseases who underwent LLR were preoperatively enrolled, and divided into two groups according to whether using a 3D-printed dry-laboratory model (3D vs. control group). Clinical variables were assessed and complications were graded by the Clavien-Dindo classification. The Comprehensive Complication Index (CCI) scores were calculated and compared for each patient. Multivariable analysis was performed to determine the risk factors of postoperative complications.

**Results:**

Sixty-two patients with complex hepatobiliary diseases underwent the precise planning of LLR. Among them, thirty-one patients acquired the guidance of a 3D-printed dry-laboratory model, and others were only guided by traditional enhanced CT or MRI. The results showed no significant differences between the two groups in baseline characters. However, compared to the control group, the 3D group had a lower incidence of intraoperative blood loss, as well as postoperative 30-day and major complications, especially bile leakage (all *P* < 0.05). The median score on the CCI was 20.9 (range 8.7–51.8) in the control group and 8.7 (range 8.7–43.4) in the 3D group (mean difference, -12.2, *P* = 0.004). Multivariable analysis showed the 3D model was an independent protective factor in decreasing postoperative complications. Subgroup analysis also showed that a 3D model could decrease postoperative complications, especially for bile leakage in patients with intrahepatic cholelithiasis.

**Conclusion:**

The 3D-printed models can help reduce postoperative complications. The 3D-printed models should be recommended for patients with complex hepatobiliary diseases undergoing precise planning LLR.

**Supplementary Information:**

The online version contains supplementary material available at 10.1186/s12893-024-02441-z.

## Introduction

Minimally invasive and precision therapies comprise the new direction of surgery in the 21st century. Since the first laparoscopic liver tumor resection was reported by Reich et al. in 1991 [1], laparoscopic technology has become increasingly widely used in the diagnosis and treatment of hepatobiliary diseases. Multiple meta-analyses have shown that precise liver resection for primary liver cancer is correlated with less trauma, faster recovery, and better prognosis than conventional liver resection. However, there are still great difficulties in applying minimally invasive and precise treatment for complex hepatobiliary diseases because it is difficult to expose certain parts of the liver or remove complex hepatobiliary lesions under laparoscopy. In addition, the liver has a complex anatomical structure and may bleed easily, necessitating the conversion to open surgery. As of 2016, more than 9,000 cases of laparoscopic liver resection had been reported worldwide, with approximately 30% of these cases involving extensive and complex hepatectomies [2, 3]. However, the rate of conversion to open surgery in complex liver resections is almost 40% and postoperative complications are about 33.4 − 46.9% [4–7], making it difficult to fully achieve precise treatment [8]. Therefore, there is an urgent need for the development of precise planning laparoscopic surgery for special and complex hepatobiliary diseases [9].

Precise liver resection requires three-dimensional (3D) reconstruction technology, 3D printing technology, and intraoperative navigation technology [10]. Minimally invasive and precise anatomical liver resection helps the surgeon to completely remove the lesion, effectively control the bleeding, retain the maximum remaining liver function, reduce the degree of trauma, and accelerate the patient’s recovery. However, safe and effective radical liver resection requires detailed preoperative planning, simulation training, and laparoscopic navigation technology to accurately identify the appropriate surgical margins, blood vessels, and bile ducts. Preoperative planning has long relied on imaging modalities such as CT and MRI, especially 3D reconstruction imaging of liver tumors [11–13]. However, 3D images also have shortcomings, such as angular deviations which will make it difficult to relocate the tumor intraoperatively. These shortcomings can be reduced using 3D-printed models that visually show the range of the liver and the liver tumor accurately, and can detect the normal liver volume and residual liver volume through a computer-assisted system. Thus, 3D-printed models help surgeons to comprehensively evaluate liver function and accurately perform liver resection while reducing surgical risk [14]. Multiple studies have shown that 3D printing technology is helpful in preoperative surgical planning, promoting effective preoperative communication with patients and their families, and improving the precise surgical treatment of liver diseases [15].

The current study aims to evaluate the application value of preoperative 3D-printed dry-laboratory models in the precise planning of laparoscopic surgery for complex hepatobiliary diseases. By comparing with traditional enhanced CT or MRI, we aim to clarify the improvement effect of 3D printing models on intraoperative and postoperative complications. The successful application of this model aims to assist clinicians in decision-making, especially in helping clinicians better perform high-difficulty surgeries such as precise planning of laparoscopic surgery for complex hepatobiliary diseases, and to benefit the patients.

## Patients and methods

### Patients

Eligible patients diagnosed with intrahepatic cholelithiasis, hepatocellular carcinoma (HCC), or intrahepatic cholangiocarcinoma (ICC) were consecutively and prospectively enrolled between June 2018 and August 2023. The inclusion criteria were preoperative imaging revealing complex disease requiring extensive resection of liver lesions or lesions located in special sites, no distant tumor metastasis, and a liver function of Child-Pugh grade A or B. Complex hepatectomy was determined according previous studies reported [16–19], including (1) extensive left or right hemi-hepatectomy, (2) meso-hepatectomy (involving S4a and/or S8), (3) more than 3 segments, (4) special sites (S1), (5) near the first or second portal of the liver (< 1 cm). The exclusion criteria were preoperative chemoradiotherapy or severe cardiopulmonary disease. A total of 62 patients were divided into two groups: the 3D group underwent a flat layer scan of their CT or MRI [11] data, with a slice thickness of 1 mm, to generate 3D reconstructed images depicting the lesion location, critical vessels, bile ducts, and the targeted resection area. The pertinent data were then transmitted to the 3D printer in a stereolithography format, which was specifically tailored for 3D printing purposes. Using this data, a life-size 3D liver model was printed. The evaluation metrics encompassed operative duration, intraoperative bleeding and blood transfusion requirements, postoperative complications, and hospital stay duration within each group. The ethics committee of Zhejiang Provincial Peoples Hospital granted approval for the study protocol, and all participants provided informed consent to participate in the study.

### The 3D Printing process

**Fig. 1:**
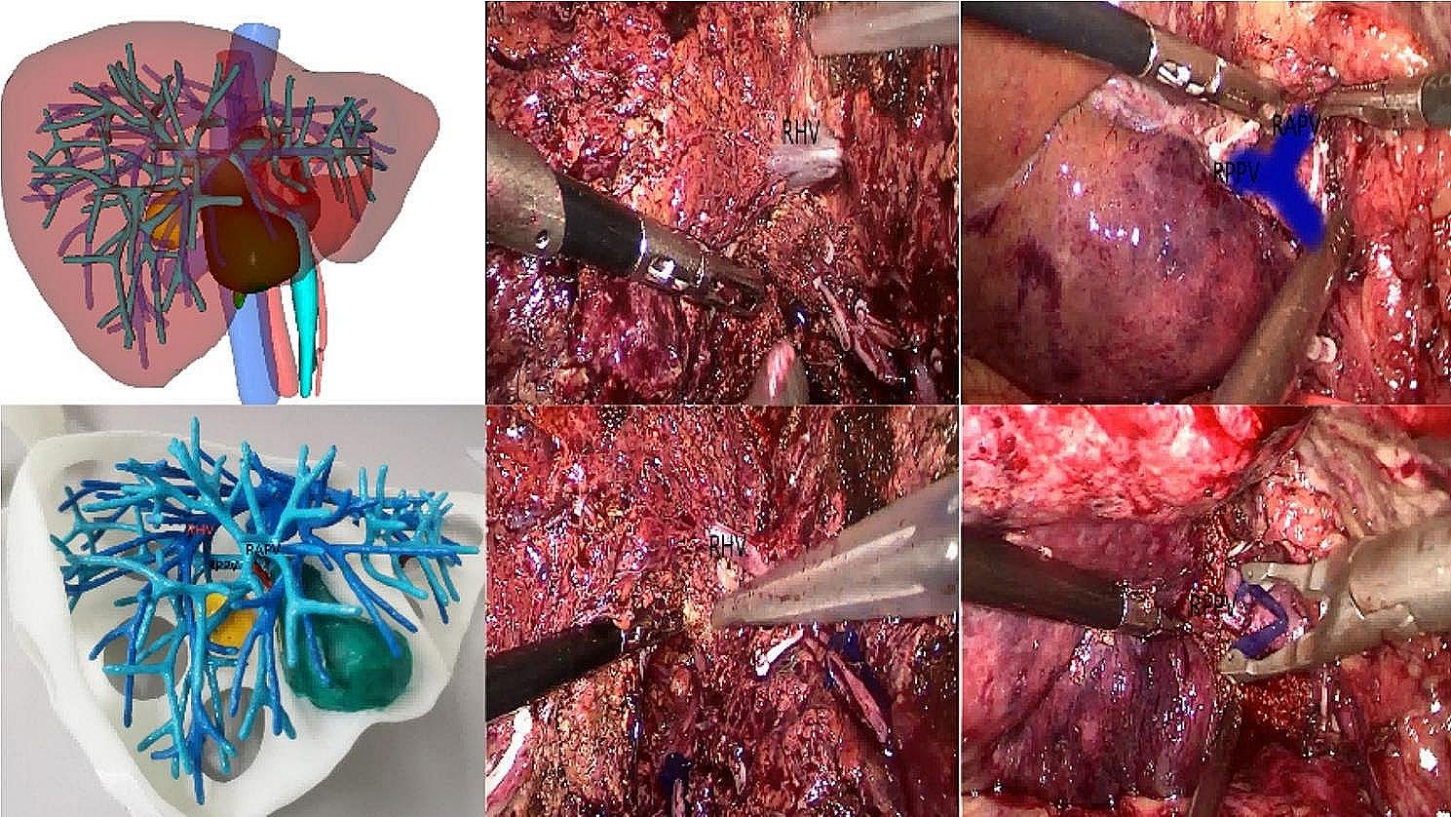
The application of the 3D reconstructed images and the 3D print model for intraoperative navigation. RHV, right hepatic vein; RAPV, right anterior portal vein; RPPV, Right posterior portal vein

The hepatic segmentation and 3D virtual reconstruction were carried out utilizing the E3D digital medical modeling software V17.06, developed by the Central and Southern E3D Digital Medical and Virtual Reality Research Center in China [20]. This process was grounded on the patients’ CT Dicom (Digital Imaging and Communications in Medicine) data, as depicted in Fig. 1. Subsequently, the positioning of lesions alongside intricate vascular and biliary structures was meticulously analyzed and planned using Cura 4.4.1, an open-source slicing software from Ulitmaker in the United States. This analysis culminated in the generation of G code specific to SLA (Stereo Lithography Appearance), which was recognized by the SL600 printer manufactured by ZhongRuiZhiChuang3D Technology Co., LTD. in Suzhou, China. This printer was utilized to produce physical liver models. The material used for these models comprised photosensitive resin, specifically ZR680 from ZhongRuiZhiChuang3D Technology Co., LTD. This material exhibited a bending strength of 66 ∼ 73 MPa and a fracture elongation rate of 10%∼15%. The liquid photosensitive material, after being degassed, was solidified and printed layer by layer under the control of an ultraviolet system. Notably, only the lesions along with blood vessels, bile ducts, and their branches with a diameter greater than 2 mm were printed, excluding extrahepatic parenchyma. The surface of the model was hollowed out, creating apertures with a diameter of 45–50 mm. Following curing with a UV mercury lamp and coloring in a post-processing box, the 3DP liver model was successfully completed.

### Clinicopathological characteristics and Perioperative Morbidity

All the included clinicopathological characteristics were prospectively collected from the medical records system at Zhejiang Provincial Peoples Hospital, including gender, age, American Society of Anesthesiologists (ASA) score, Child-Pugh, alanine aminotransferase (ALT), aspartate transaminase (AST), international normalized ratio (INR), white blood cell (WBC), C-reactive protein (CRP), alpha-fetoprotein (AFP), carbohydrate antigen 19 − 9 (CA19-9), carcinoembryonic antigen (CEA), type of disease, disease location, proximity to the first or second hepatic portal, time of surgery, major hepatectomy, intraoperative bleeding, inoperative blood transfusion, conversed to open surgery, postoperative hospital stays, and postoperative 30-day mortality and morbidity. Comorbid illnesses were determined as consisting of obesity, diabetes mellitus, chronic cardiovascular or obstructive pulmonary disease, and renal dysfunction history. Major hepatectomy refers to the resection of more than 3 liver segments [21]. Perioperative morbidity and mortality were collected, including post hepatectomy liver failure (PHLF) [22], bleeding, blood transfusion, bile leakage, pneumonia, hydrothorax or seroperitoneum, and puncture drainage. Hospital stays were calculated from surgery to discharge. Clavien–Dindo I-II was set as minor morbidity and Clavien–Dindo III-V was set as major morbidity [23]. Comprehensive complication index (CCI) scores are obtained by entering all patients’ complications into the Clavien-Dindo system classification using the online calculator (www.cci-calculator.com).

### Statistical analysis

The categorical variables were utilized to represent all the data. The Levene’s test was employed to assess the homogeneity of variance. To compare the characteristics of the two groups, the Fisher’s exact test was applied. The statistical analysis was conducted using the SPSS software, version 22.0, which was developed by SPSS Inc., Chicago, IL, USA. Statistical significance was determined based on a two-tailed P value that was less than 0.05.

## Results

### Characteristics of patients

Sixty-two patients with complex hepatobiliary diseases who underwent the precise planning of LLR were included. Most of the patients were male (66%) and Child-Pugh A (90%). Among them, thirty-one patients acquired the guidance of a 3D-printed dry-laboratory model, and the others were only guided by traditional enhanced CT or MRI. There were no differences between the two groups in baseline characteristics including age, ASA score, type of disease, tumor, or disease location (all *P* > 0.05) (Table 1).

**Table 1:** Compare the demographic and oncological characteristics between the two groups

*N*, %	3D group (*n* = 31)	Control group (*n* = 31)	P*
Gender, male/ female	22 (71.0)/ 9 (29.0)	19 (61.3)/ 12 (38.7)	0.592
Age, < 60/ ≥ 60 year	18 (58.1)/ 13 (41.9)	14 (45.2)/ 17 (54.8)	0.446
ASA score, 1/ ≥ 2	18 (58.1)/ 13 (41.9)	12 (38.7)/ 19 (61.3)	0.204
Comorbid illness, with/ without	16 (51.6)/ 15 (48.4)	11 (35.5)/ 20 (64.5)	0.306
Child-Pugh, A/B	28 (90.0)/3 (10.0)	28 (90.0)/3 (10.0)	1.000
ALT level, < 80/ ≥ 80 U/L	25 (80.6)/ 6 (19.4)	24 (77.4)/7 (22.6)	1.000
AST level, < 80/ ≥ 80 U/L	22 (71.0)/9 (29.0)	19 (61.3)/12 (38.7)	0.592
INR, ≤ 1.20/ > 1.20	28 (90.3)/3 (9.7)	26 (86.9)/5 (16.1)	0.707
WBC, ≤ 9.5/ > 9.5*10^9^/L	27 (87.1)/ 4 (9.7)	26 (86.9)/5 (16.1)	0.731
CRP, ≤ 10/ > 10 mg/L	21 (67.7)/ 10 (32.3)	22 (71.0)/ 9 (29.0)	1.000
Type of disease, benign / malignant	11 (35.5)/20 (64.5)	8 (25.8)/ 23 (74.2)	0.582
Disease location	13 (41.9)/ 12 (38.7)/ 6 (19.4)	7 (22.6)/ 14 (45.2)/ 10 (32.3)	0.228
Left/ Right/ Median hepatic lobe			
Proximity to the first hepatic portal, < 1/ ≥1 cm	7 (22.6)/ 24 (77.4)	12 (38.7)/ 19 (61.3)	0.270
Proximity to the second hepatic portal, < 1/ ≥1 cm	8 (25.8)/ 23 (74.2)	14 (45.2)/ 17 (54.8)	0.184

* Fisher’s exact test was used for classification variables. ASA, American Society of Anesthesiologists; ALT, alanine aminotransferase; AST, aspartate transaminase, INR, international normalized ratio; WBC, white blood cell; CRP, C-reactive protein

### Intraoperative Variables and postoperative complications

Two patients suffered a conversion to open surgery in the 3D group, due to the heavy adhesion in the abdominal cavity after the first operation (Table 2). Five patients suffered a conversion to open surgery in the control group, among them, three of the patients were due to heavy intraperitoneal adhesion, one due to intraoperative bleeding, and one due to inadequate exposure of the visual field (*P* = 0.425). Of note, intraoperative bleeding volume in the 3D group was significantly lower than the control group (12.9 vs. 38.7, *P* = 0.040). No patient died within 90 days after LLR. The median score on the CCI was 20.9 (range 8.7–51.8) in the control group and 8.7 (range 8.7–43.4) in the 3D group (mean difference, -12.2, *P* = 0.004).The incidence of postoperative 30-day morbidity was 46.8% (3D group 29.0% vs. control group 64.5%, *P* = 0.010). Of these, 21.0% was minor morbidity (3D group 16.1% vs. control group 25.8%, *P* = 0.534) and 25.8% were major morbidity (3D group 12.9% vs. control group 38.7%, *P* = 0.040). Furthermore, the incidence of bile leakage in the 3D group was lower than in the control group (6.5% vs. 35.5%, *P* = 0.011). In addition, the median hospital stay after LLR was 9 (range 7–22) days in the 3D group, and 11 (range 10–30) days (*P* = 0.433).

**Table 2:** Compare the intraoperative variables and postoperative complications between the two groups

*N*, %	3D group (*n* = 31)	Control group (*n* = 31)	P*
Time of surgery, ≥ 180 min	14 (45.2)	18 (58.1)	0.446
Major hepatectomy, ≥ 3 segments	23 (74.2)	27 (87.1)	0.335
Intraoperative bleeding, ≥ 600mL	4 (12.9)	12 (38.7)	**0.040**
Inoperative blood transfusion, yes	5 (16.1)	8 (25.8)	0.534
Conversed to open surgery, yes	2 (6.5)	5 (16.1)	0.425
Postoperative hospital stays, days	9 (7–22)	11 (10–30)	0.433
Postoperative 30-day mortality	0 (0)	0 (0)	1.000
Postoperative 30-day morbidity	9 (29.0)	20 (64.5)	**0.010**
Minor (Clavien-Dindo I-II)	5 (16.1)	8 (25.8)	0.534
Major (Clavien-Dindo III-V)	4 (12.9)	12 (38.7)	**0.040**
hepatic failure	4 (12.9)	6 (19.4)	0.731
postoperative hemorrhage	2 (6.5)	3 (9.7)	1.000
bile leakage	2 (6.5)	11 (35.5)	**0.011**
pneumonia	7 (22.6)	8 (25.8)	1.000
Chest or abdominal fluid	4 (12.9)	5 (16.1)	1.000
puncture drainage	6 (19.4)	8 (25.8)	0.762

### Independent risk factors of postoperative complications

To determine the independent risk factors, variables with a univariable *P* < 0.1 were entered into the multivariable analyses forward stepwise (Table 3). The results of the multivariable analysis showed that precise planning LLR based on 3D-printed dry-laboratory models can reduce perioperative complications for patients with complex hepatobiliary diseases (OR 0.677, 95%CI 0.084–0.915, *P* = 0.035).

**Table 3:** Univariable and multivariable logistic regression analyses of risk factors associated with postoperative mortality for patients with complex hepatobiliary diseases after laparoscopic liver resection

Variables	UV OR (95% CI)	*P*	MV OR (95% CI)	P*
Gender, male vs. female	7.700 (1.555–38.137)	0.012	NS	
Age, > 60 vs. ≤ 60 years	3.621 (0.737–17.802)	0.113		
ASA score, > 2 vs. ≤ 2	5.160 (1.025–25.981)	0.047	NS	
Comorbid illness, yes vs. no	3.099 (0.799–12.024)	0.102		
Child-Pugh grade, B vs. A	1.904 (0.113–31.999)	0.655		
ALT level, > 80 vs. ≤ 80 U/L	3.502 (0.340-36.098)	0.292		
AST level, > 80 vs. ≤ 80 U/L	2.293 (0.377–13.952)	0.368		
INR, > 1.20 vs. ≤ 1.20	5.743 (0.496–6.505)	0.162		
WBC, > 9.5 vs. ≤ 9.5 *10^9^/L	3.618 (0.167-4.000)	0.299		
CRP, ≤ 10/ > 10 mg/L	1.480 (0.167–13.111)	0.725		
Disease, benign / malignant	1.367 (0.408–4.581)	0.613		
Disease location, Left hepatic lobe	Reference		Reference	
Right hepatic lobe	1.499 (1.134–2.865)	0.031	1.117 (1.101–1.727)	0.028
Median hepatic lobe	1.795 (1.196–3.224)	0.008	1.526 (1.112–2.472)	0.016
Proximity to the first hepatic portal, < 1 vs. ≥ 1 cm	4.612 (1.310-16.239)	0.017	3.711 (1.074–8.825)	0.038
Proximity to the second hepatic portal, < 1 vs. ≥ 1 cm	1.464 (0.449–4.771)	0.527		
Major hepatectomy, yes vs. no	3.991 (1.167–13.642)	0.027	1.296 (1.088–2.840)	0.036
Intraoperative blood loss, > 600 vs. ≤ 600 mL	2.092 (0.460–9.510)	0.339		
Intraoperative blood transfusion, yes vs. no	2.323 (0.427–12.648)	0.330		
Operation time, ≥ 180 vs. <180 min	1.177 (0.267–5.186)	0.830		
Guided by 3D model, yes vs. no	0.353 (0.102–0.916)	0.049	0.677 (0.084–0.915)	0.035

* *P* < 0.1 in univariable analyses were entered into multivariable analyses. ASA, American Society of Anesthesiologists; ALT, alanine aminotransferase; AST, aspartate transaminase, INR, international normalized ratio; WBC, white blood cell; CRP, C-reactive protein. MV, multivariable; NA, not available; OR, odds ratio; UV, univariable; NS, no significance

### Subgroup Analysis

Subgroup analysis was performed according to patients with benign or malignant disease. Among the included patients, 43 (69%) patients were diagnosed with primary liver cancer (HCC, 19 patients and ICC, 24 patients), and 19 (31%) patients were diagnosed with intrahepatic cholelithiasis. For patients with liver cancer, there were no differences between the two groups in baseline characteristics (Supplement Tables 1 and 2). What’s more, 3D-printed dry-laboratory models can reduce the incidence of perioperative complications (30.0% vs. 60.9%, *P* = 0.042) (Table 4). For patients with intrahepatic cholelithiasis, there were also no differences between the two groups in baseline characteristics (Supplement Table 3). Of note, the results showed that 3D-printed dry-laboratory models can significantly reduce the incidence of major complications (9.1% vs. 62.5%, *P* = 0.041), especially the incidence of bile leakage (1 9.1% vs. 62.5%, *P* = 0.041) (Table 5).

**Table 4:** Compare the intraoperative variables and postoperative complications between the two groups for patients with liver cancer

*N*, %	3D group (*n* = 20)	Control group (*n* = 23)	P*
Time of surgery, ≥ 180 min	9 (45.0)	12 (52.2)	0.763
Major hepatectomy, ≥ 3 segments	14 (70.0)	20 (78.3)	0.318
Intraoperative bleeding, ≥ 600mL	4 (20.0)	11 (47.8)	0.107
Inoperative blood transfusion, yes	5 (25.0)	7 (30.4)	0.745
Conversed to open surgery, yes			
Postoperative hospital stays, days	8 (4–30)	9 (5–41)	0.535
Postoperative 30-day complications	6 (30.0)	14 (60.9)	**0.042**
Minor (Clavien-Dindo I-II)	4 (20.0)	5 (21.7)	1.000
Major (Clavien-Dindo III-V)	3 (15.0)	8 (34.8)	0.175
hepatic failure	3 (15.0)	5 (21.7)	0.704
postoperative hemorrhage	3 (15.0)	3 (13.0)	0.610
bile leakage	1 (5.0)	6 (26.1)	0.070
pneumonia	4 (20.0)	5 (21.7)	1.000
Chest or abdominal fluid	3 (15.0)	5 (21.7)	0.704
puncture drainage	5 (25.0)	4 (17.4)	0.711

**Table 5:** Compare the intraoperative variables and postoperative complications between the two groups for patients with intrahepatic cholelithiasis

*N*, %	3D group (*n* = 11)	Control group (*n* = 8)	P*
Time of surgery, ≥ 180 min	5 (45.5)	6 (75.0)	0.352
Major hepatectomy, ≥ 3 segments	9 (81.8)	7 (87.5)	1.000
Intraoperative bleeding, ≥ 600mL	0 (0)	1 (12.5)	0.421
Inoperative blood transfusion, yes	0 (0)	1 (12.5)	0.421
Conversed to open surgery, yes	1 (9.1)	2 (25.0)	0.546
Postoperative hospital stays, days	10 (6–16)	12 (6–20)	0.342
Postoperative 30-day morbidity	3 (27.3)	6 (75.0)	0.070
Minor (Clavien-Dindo I-II)	2 (18.2)	2 (25.0)	1.000
Major (Clavien-Dindo III-V)	1 (9.1)	5 (62.5)	**0.041**
hepatic failure	1 (9.1)	1 (12.5)	1.000
postoperative hemorrhage	1 (9.1)	0 (0)	1.000
bile leakage	1 (9.1)	5 (62.5)	**0.041**
pneumonia	3 (27.3)	3 (37.5)	1.000
Chest or abdominal fluid	1 (9.1)	0 (0)	1.000
puncture drainage	1 (9.1)	4 (50.0)	0.071

## Discussion

In the present study, sixty-two patients with complex hepatobiliary diseases who underwent the precise planning of LLR were included. There were no differences between the two groups in baseline characteristics. Whereas, analysis of intraoperative variables revealed that the 3D group had significantly reduced blood loss. In addition, the analysis of postoperative complications showed that the overall incidence of complications and serious complications in the 3D group was significantly reduced. Of note, precise planning LLR based on 3D-printed dry-laboratory models can reduce postoperative complications for patients with complex hepatobiliary diseases (OR 0.677, 95%CI 0.084–0.915, *P* = 0.035). In other words, precise planning LLR based on 3D-printed dry-laboratory models can decrease nearly 32% risk of postoperative complications. The results of the subgroup analysis further confirmed the conclusion.

It is complex and difficult to resect lesions in complex parts of the liver or lesions involving multiple liver segments [17]. Patients with such special or complex liver lesions may develop postoperative liver failure or poor prognosis due to insufficient remaining functional liver or severe complications, resulting in surgical failure. Therefore, intraoperative precise liver resection and avoidance of important vascular bile duct injury are very important [24, 25]. Currently, most evaluations of preoperative liver function and vascular location are based on imaging modalities such as CT and MRI, especially 3D reconstructed images of liver tumors. However, the lack of real contact in the 3D reconstructed images may lead to inaccurate preoperative evaluation and biased intraoperative navigation, especially in complex hepatobiliary diseases. Igami et al. suggested that there are individual differences in the 3D structure constructed based on two-dimensional images, while the spatial relationship between the tumor lesions and the surrounding tissue in the 3D-printed liver model is consistent [26]. The 3D printing technique transforms 3D reconstructed images into actual objects, enabling clinicians to directly view the complex intrahepatic blood vessels and bile ducts, improve the understanding of the complex anatomy of the liver, and improve the accuracy of liver resection [27]. The 3D-printed model can make up for the shortcomings of 3D images and enables the assessment of the normal liver volume and residual liver volume through a computer-aided system, which gives a more comprehensive assessment of liver function and contributes to the safe performance of liver surgery.

In the diagnosis and treatment of liver cancer, 3D printing technology has been used to preoperatively clarify the spatial relationship between intrahepatic vessels and tumors, which reduces the risk of intraoperative vascular injury, reduces the amount of bleeding, and shortens the operative time. Thus, 3D-printed models make up for the limitations of “spatial imagination“ [28]. As we can see from Fig. 1, the use of preoperative 3D reconstructed images may lead to liver resection errors in real-time navigation during liver resection, with the degree of error related to the experience of the surgeon. In contrast, the 3D model enables the surgeon to measure the distances between points and conduct accurate tangent positioning to obtain R0 resection. Furthermore, the surgeon can obtain the most intuitive sense of direction by continuously comparing the physical objects with the 3D model during surgery; the surgeon can adjust the spatial position of the 3D model in real-time according to the specific situation to conduct accurate intraoperative repositioning and clarify the spatial location of the lesion to achieve the effect of real-time navigation, thus reducing the surgical risk. The 3D-printed model is particularly useful for patients requiring extensive liver resection with vascular or biliary invasion. Detailed information on vascular and bile duct anatomy provided by 3D-printed models can even replace intraoperative ultrasonography or cholangiography, which is crucial in reducing the operative time and complications. Fang et al. constructed 3D visualization models of 56 patients to clearly show the anatomy of blood vessels, tumor location, and size, and the relationship between blood vessels and tumor to assist in surgical planning [29]. The 3D-printed model of eleven patients requiring complex liver resection was identical to the anatomy seen during surgery, enabling complete tumor resection. The 3D-printed model helps improve the understanding of the morphology of the lesion and the surrounding normal tissue and important structures (such as bile ducts and blood vessels) and provides intraoperative real-time navigation to enable the preservation of more normal liver tissue and reduce the occurrence of complications such as postoperative liver failure [30]. In summary, 3D-printed models reduce surgical risks, and improve the safety, effectiveness, and accuracy of surgery [1, 31].

Intrahepatic cholelithiasis is another type of liver surgery that is difficult to perform. Due to chronic inflammation, the liver atrophy, blood vessels, and bile ducts are located in a variable position. In addition, to achieve a complete cure for intrahepatic cholelithiasis, it is necessary to remove as much of the bile duct containing stones as possible, which requires preserving as much normal liver tissue as possible. In our study, subgroup analysis showed that in patients with intrahepatic cholelithiasis, the incidence of postoperative bile leakage in the 3D group was significantly lower than that in the control group. We speculate that this is because patients with intrahepatic bile duct stones often have stenosis, inflammation, local expansion, and other lesions, leading to intraoperative bile duct anatomy difficulties and possible bile leakage. We also found that the surgical plan changed in some cases after the application of the 3D-printed model in preoperative planning. One such case involved a patient with intrahepatic cholelithiasis who was scheduled to undergo laparoscopic resection of the right anterior liver lobe (segment V/VIII) based on imaging data and 3D reconstructed images. After preoperative planning with the 3D-printed model, the surgical strategy was changed to a more accurate laparoscopic resection of liver segment V, as the model showed that the anatomical resection of liver segment V was sufficient to remove the lesion while retaining the maximum amount of liver tissue.

There are also some limitations in this study. Firstly, the retrospective study had a fixed selection bias, although we used the preoperative method to reduce the impact of selection bias on outcomes. Secondly, all the LLR was performed by experienced surgeons. LLR for complex HCC requires a learning curve for surgeons. Thirdly, intrahepatic cholelithiasis, HCC, and ICC were enrolled in this study. Further validation, especially multicenter randomized controlled trials for a single disease, still needed to be conducted.

## Conclusion

The 3D-printed models can help reduce postoperative complications in patients undergoing complex liver resection, especially in patients with intrahepatic cholelithiasis. Further studies are warranted to confirm the present findings.

### Electronic supplementary material

Below is the link to the electronic supplementary material.

Supplementary Material 1

## Data Availability

The datasets used and analyzed during the current study are available from the corresponding author upon reasonable request.

## Abbreviations

LLR: laparoscopic liver resection
HCC: hepatocellular carcinoma
3D: three-dimensional
ASA: American Society of Anesthesiologists
ALT: alanine aminotransferase
AST: aspartate transaminase
ALT: alanine aminotransferase
INR: international normalized ratio
WBC: white blood cell
CRP: C-reactive protein
AFP: alpha-fetoprotein
CA19-9: carbohydrate antigen 19 − 9
CEA: carcinoembryonic antigen
HCC: hepatocellular carcinoma
ICC: intrahepatic cholangiocarcinoma
UV: univariable
MV: multivariable
CI: confidence interval
OR: odds ratio
NS: no significance
