# Supplementary material for: Precise planning based on 3D-printed dry-laboratory models can reduce perioperative complications of laparoscopic surgery for complex hepatobiliary diseases: a preoperative cohort study
Source: BMC Surg. 2024 May 11;24:148. doi: 10.1186/s12893-024-02441-z (PMC11088180; doi:10.1186/s12893-024-02441-z)
Supplement: Supplementary file 1 — Supplementary Material 1 [file 12893_2024_2441_MOESM1_ESM.docx]

**Supplement Table 1**: Compare the demographic and oncological characteristics between the two groups in patients with liver cancer

| **N, %** | **3D group**  **(n = 20)** | **Control group**  **(n = 23)** | **P*** |
| --- | --- | --- | --- |
| Gender, male/ female | 2 (10.0)/ 18 (90.0) | 6 (26.1)/ 17 (73.9) | 0.250 |
| Age, <60/ ≥ 60 year | 11 (55.0)/ 9 (45.0) | 9 (39.1)/ 14 (60.9) | 0.366 |
| ASA score, 1/ ≥ 2 | 9 (45.0)/ 11 (55.0) | 8 (34.8)/ 15 (65.2) | 0.545 |
| Comorbid illness, with/ without | 11 (55.0)/ 9 (45.0) | 9 (39.1)/ 14 (60.9) | 0.366 |
| Child-Pugh， B/ A | 2 (10.0)/ 18 (90.0) | 2 (8.7)/ 21 (91.3) | 1.000 |
| ALT level, <80/ ≥ 80 U/L | 15 (75.0)/ 5 (25.0) | 18 (78.3)/ 5 (21.7) | 1.000 |
| AST level, <80/ ≥ 80 U/L | 13 (65.0)/ 7 (35.0) | 14 (60.9)/ 9 (39.1) | 1.000 |
| INR, ≤ 1.20/ > 1.20 | 17 (85.0)/ 3 (15.0) | 20 (87.0)/ 3 (13.0) | 1.000 |
| WBC, ≤ 9.5/ > 9.5*10^9^/L | 20 (100.0)/ 0 (0) | 22 (95.7)/ 1 (4.3) | 1.000 |
| CRP, ≤ 10/ > 10 mg/L | 19 (95.0)/ 1 (5.0) | 21 (91.3)/ 2 (8.7) | 1.000 |
| AFP，< 20/ ≥20 ng/mL | 11 (55.0)/ 9 (45.0) | 14 (60.9)/ 9 (39.1) | 0.763 |
| CA19-9，< 40/ ≥ 40 ng/mL | 12 (60.0)/ 8 (40.0) | 12 (52.2)/ 11 (47.5) | 0.760 |
| CEA，< 10/ ≥ 10 ng/mL | 1 (5.0)/ 19 (95.0) | 2 (8.7)/ 21 (91.3) | 0.637 |
| Disease, HCC / ICC | 7 (35.0)/ 13 (65.0) | 12 (52.2)/ 11 (47.8) | 0.359 |
| Disease location | 8 (40.0)/ 8 (40.0)/  4 (20.0) | 6 (26.1)/ 12 (52.2)/  5 (21.7) | 0.609 |
| Left/ Right/ Median hepatic lobe |  |  |  |
| Proximity to the first hepatic portal, < 1/ ≥1 cm | 3 (15.0)/ 17 (85.0) | 8 (34.8)/ 15 (65.2) | 0.175 |
| Proximity to the second hepatic portal, < 1/ ≥1 cm | 5 (25.0)/ 15 (75.0) | 10 (43.5)/ 13 (56.5) | 0.336 |

* Fisher's exact test was used for classification variables. ASA, American Society of Anesthesiologists; ALT, alanine aminotransferase; AST, aspartate transaminase, INR, international normalized ratio; WBC, white blood cell; CRP, C-reactive protein; AFP, alpha fetoprotein; CA19-9, carbohydrate antigen 19-9; CEA, carcinoembryonic antigen; HCC, hepatocellular carcinoma; ICC, intrahepatic cholangiocarcinoma.

**Supplement Table 2**: Detailed information on the diagnosis of tumor patients and the size of the largest tumor lesion diameter.

| **Patients, diameter (diagnosis)** | **3D group (n = 20)** | **Control group (n = 23)** | **P*** |
| --- | --- | --- | --- |
| No.1 | 78 (HCC) | 70 (HCC) |  |
| No.2 | 75 (HCC) | 70 (HCC) |  |
| No.3 | 63 (HCC) | 59 (HCC) |  |
| No.4 | 62 (HCC) | 50 (HCC) |  |
| No.5 | 59 (HCC) | 35 (HCC) |  |
| No.6 | 45 (HCC) | 28 (HCC) |  |
| No.7 | 34 (HCC) | 70 (HCC) |  |
| No.8 | 70 (ICC) | 64 (HCC) |  |
| No.9 | 65 (ICC) | 62 (HCC) |  |
| No.10 | 55 (ICC) | 60 (HCC) |  |
| No.11 | 35 (ICC) | 38 (HCC) |  |
| No.12 | 50 (ICC) | 32 (HCC) |  |
| No.13 | 47 (ICC) | 59 (ICC) |  |
| No.14 | 73 (ICC) | 62 (ICC) |  |
| No.15 | 46 (ICC) | 35 (ICC) |  |
| No.16 | 49 (ICC) | 36 (ICC) |  |
| No.17 | 75 (ICC) | 48 (ICC) |  |
| No.18 | 48 (ICC) | 58 (ICC) |  |
| No.19 | 57 (ICC) | 52 (ICC) |  |
| No.20 | 45 (ICC) | 44 (ICC) |  |
| No.21 | - | 53 (ICC) |  |
| No.22 | - | 60 (ICC) |  |
| No.23 | - | 64 (ICC) |  |
| Median HCC | 62 (34-78) | 60 (28-70) | 0.399 |
| Median ICC | 50 (35-75) | 50 (35-64) | 0.521 |

HCC, hepatocellular carcinoma; ICC, intrahepatic cholangiocarcinoma. Median diameter was presented as median (range), and compared by the rank sum test.

**Supplement Table 3**: Compare the demographic and oncological characteristics between the two groups in patients with intrahepatic cholelithiasis

| **N, %** | **3D group**  **(n = 11)** | **Control group**  **(n = 8)** | **P*** |
| --- | --- | --- | --- |
| Gender, male/ female | 7 (63.6)/ 4 (36.4) | 6 (75.0)/ 2 (25.0) | 1.000 |
| Age, <60/ ≥ 60 year | 4 (36.4)/ 7 (62.5) | 3 (37.5)/ 5 (62.5) | 1.000 |
| ASA score, 1/ ≥ 2 | 9 (81.8)/ 2 (18.2) | 4 (50.0)/ 4 (50.0) | 0.319 |
| Comorbid illness, with/ without | 5 (45.5)/ 6 (54.5) | 2 (25.0)/ 6 (75.0) | 0.633 |
| Child-Pugh，A/B | 10 (90.9)/ 1 (9.1) | 7 (87.5)/ 1 (12.5) | 1.000 |
| ALT level, <80/ ≥ 80 U/L | 10 (90.9)/ 1 (9.1) | 6 (75.0)/ 2 (25.0) | 0.546 |
| AST level, <80/ ≥ 80 U/L | 9 (81.8)/ 2 (18.2) | 5 (62.5)/ 3 (37.5) | 0.603 |
| INR, ≤ 1.20/ > 1.20 | 11 (100)/ 0 (0) | 6 (75.0)/ 2 (25.0) | 0.164 |
| WBC, ≤ 9.5/ > 9.5*10^9^/L | 7 (63.6)/ 4 (36.4) | 3 (37.5)/ 5 (62.5) | 0.370 |
| CRP, ≤ 10/ > 10 mg/L | 2 (18.2)/ 9 (81.8) | 1 (12.5)/ 7 (87.5) | 1.000 |
| Disease location | 5(45.5)/ 4 (36.4)/  2 (18.2) | 1 (12.5)/ 2 (25.0)/  5 (62.5) | 0.119 |
| Left/ Right/ Median hepatic lobe |  |  |  |
| Proximity to the first hepatic portal, < 1/ ≥1 cm | 4 (36.4)/ 7 (63.6) | 4 (50.0)/ 4 (50.0) | 0.658 |
| Proximity to the second hepatic portal, < 1/ ≥1 cm | 3 (27.3)/ 8 (72.7) | 4 (50.0)/ 4 (50.0) | 0.377 |

* Fisher's exact test was used for classification variables. ASA, American Society of Anesthesiologists; ALT, alanine aminotransferase; AST, aspartate transaminase, INR, international normalized ratio; WBC, white blood cell; CRP, C-reactive protein.
